# An immune-related gene prognostic prediction risk model for neoadjuvant chemoradiotherapy in rectal cancer using artificial intelligence

**DOI:** 10.3389/fonc.2024.1294440

**Published:** 2024-02-09

**Authors:** Pei Shu, Ning Liu, Xu Luo, Yuanling Tang, Zhebin Chen, Dandan Li, Dong Miao, Jiayu Duan, Ouying Yan, Leiming Sheng, Ganlu Ouyang, Sen Wang, Dan Jiang, Xiangbing Deng, Ziqiang Wang, Qingyun Li, Xin Wang

**Affiliations:** ^1^ Department of Radiation Oncology, Cancer Center, West China Hospital, Sichuan University, Chengdu, Sichuan, China; ^2^ Department of Abdominal Tumor Multimodality Treatment, Cancer Center, West China Hospital, Sichuan University, Chengdu, China; ^3^ Clinical Trial Center, National Medical Products Administration Key Laboratory for Clinical Research and Evaluation of Innovative Drugs, West China Hospital, Sichuan University, Chengdu, Sichuan, China; ^4^ Division of Thoracic Tumor Multimodality Treatment, Cancer Center, West China Hospital, Sichuan University, Chengdu, China; ^5^ Chengdu Institute of Computer Application, Chinese Academy of Sciences, Chengdu, China; ^6^ School of Computer Science and Technology, University of Chinese Academy of Sciences, Beijing, China; ^7^ Department of Pathology, West China Hospital, Sichuan University, Chengdu, China; ^8^ Sichuan University-University of Oxford Huaxi Joint Center for Gastrointestinal Cancer, Frontiers Science Center for Disease-Related Molecular Network, West China Hospital, Sichuan University, Chengdu, Sichuan, China; ^9^ Division of Gastrointestinal Surgery, Department of General Surgery, West China Hospital, Sichuan University, Chengdu, China; ^10^ Genecast Biotechnology Co., Ltd., Xishan District, Wuxi, Jiangsu, China

**Keywords:** artificial intelligence, prognostic model, immune related gene, rectal carcinoma, neoadjuvant chemoradiotherapy

## Abstract

**Background:**

This study aimed to establish and validate a prognostic model based on immune-related genes (IRGPM) for predicting disease-free survival (DFS) in patients with locally advanced rectal cancer (LARC) undergoing neoadjuvant chemoradiotherapy, and to elucidate the immune profiles associated with different prognostic outcomes.

**Methods:**

Transcriptomic and clinical data were sourced from the Gene Expression Omnibus (GEO) database and the West China Hospital database. We focused on genes from the RNA immune-oncology panel. The elastic net approach was employed to pinpoint immune-related genes significantly impacting DFS. We developed the IRGPM for rectal cancer using the random forest technique. Based on the IRGPM, we calculated prognostic risk scores to categorize patients into high-risk and low-risk groups. Comparative analysis of immune characteristics between these groups was conducted.

**Results:**

In this study, 407 LARC samples were analyzed. The elastic net identified a signature of 20 immune-related genes, forming the basis of the IRGPM. Kaplan−Meier survival analysis revealed a lower 5-year DFS in the high-risk group compared to the low-risk group. The receiver operating characteristic (ROC) curve affirmed the model’s robust predictive capability. Validation of the model was performed in the GSE190826 cohort and our institution’s cohort. Gene expression differences between high-risk and low-risk groups predominantly related to cytokine−cytokine receptor interactions. Notably, the low-risk group exhibited higher immune scores. Further analysis indicated a greater presence of activated B cells, activated CD8 T cells, central memory CD8 T cells, macrophages, T follicular helper cells, and type 2 helper cells in the low-risk group. Additionally, immune checkpoint analysis revealed elevated PDCD1 expression in the low-risk group.

**Conclusions:**

The IRGPM, developed through random forest and elastic net methodologies, demonstrates potential in distinguishing DFS among LARC patients receiving standard treatment. Notably, the low-risk group, as defined by the IRGPM, showed enhanced activation of adaptive immune responses within the tumor microenvironment.

## Introduction

Colorectal cancer is the third most common cancer and the second leading cause of cancer-related mortality worldwide (1). Locally advanced rectal cancer (LARC) constitutes up to 15% of all colorectal cancer cases (2). For LARC, neoadjuvant chemoradiotherapy (nCRT) followed by total mesorectal excision (TME) and adjuvant chemotherapy is the standard treatment recommended by the National Comprehensive Cancer Network (NCCN) guidelines (3). However, rectal cancers are a widely heterogeneous group. The prognosis of patients is different even after the same standard treatment. Therefore, there is a strong need to predict the prognosis of patients treated with the standard strategy and to guide treatment alterations when necessary (4, 5).

Tumor behavior is controlled not only by the epithelial component but also by the tumor immune microenvironment (TIME) (6). Several studies have indicated that tumor-infiltrating lymphocytes are predictive of the response to treatment and prognosis (7, 8). Thus, the relationship between the TIME and treatment response/prognosis was explored to find biomarkers or to construct models that could predict the response to treatment and prognosis (9). Along with this, the TIME can be modified by radiotherapy (10). Some studies have explored the correlation between the TIME and the clinical outcome of rectal cancer patients treated with neoadjuvant radiotherapy by immunohistochemical (IHC) methods (11). The results showed that different tumor infiltrating cell subsets (CD3+ lymphocytes, CD4+ lymphocytes, CD8+ lymphocytes, etc.) or the expression level of PD-L1 were predictive factors of the treatment response or prognosis (11–14). Due to the inconsistent results of IHC, it is hoped that further findings could be derived from gene analysis. Qian et al. explored the relationship between the gene expression profile and response to nCRT in LARC, and they developed an immune gene predictive model that could predict the response to nCRT by support vector machine (SVM) (15). Therefore, the value of immune-related gene signatures closely related to the TIME in predicting prognosis is worth studying.

Machine learning (ML), a branch of artificial intelligence, has had outstanding performance in disease diagnosis, prognosis prediction, and treatment response assessment. In previous studies, only the LASSO algorithm has been used to establish a prognostic model in LARC (16, 17). Timo M. Deist et al. suggested that random forest and elastic net logistic regression yield higher discriminative performance for chemoradiotherapy outcome than other classifier families (18). Thus, we planned to generate an optimized predictor of outcome in rectal cancer patients with the random forest and elastic net logistic regression methods in this study.

In this study, we focused on immune genes in an RNA immune-oncology panel and screened immune-related genes related to prognosis. The aim of this study was to construct an immune-related gene prognostic model (IRGPM) with the random forest and elastic net logistic regression methods for LARC patients treated with nCRT. We further characterized the immune microenvironment of patients with different prognostic risks. The results showed that IRGPM, which was established by using the random forest and elastic net methods, was a promising prognostic biomarker for patients receiving neoadjuvant treatment. Lack of effective immune infiltration in tumor microenvironment was observed in the high risk group with poorer survival.

## Materials and methods

### Data acquisition and processing

A public database was queried for patients with LARC. Only patients who received a standard strategy (neoadjuvant treatment followed by TME and adjuvant chemotherapy) and had prognostic data were selected. The gene expression data with matched clinical information were obtained from the Gene Expression Omnibus (GEO) database (https://www.ncbi.nlm.nih.gov/geo/), with accession numbers GSE87211, GSE190826 and GSE119409. Patients with missing disease-free survival (DFS) were excluded to reduce statistical bias in this analysis. The GSE87211 dataset was used as the training cohort, and the GSE190826 dataset was used as the validation cohort. These three cohorts were all included in the immune analysis. Another validation cohort was created from LARC patients at West China Hospital, Sichuan University (WCHSC). Patients with LARC who underwent standard nCRT treatment between January 2013 and January 2018 were recruited. The study was approved by the ethical committee of WCHSC. An RNA immune-oncology panel was used to detect the expression of 395 genes at the RNA level from formalin-fixed, paraffin-embedded (FFPE) tumor specimens (details are provided in **Supplementary Table S1**). The raw gene expression data in GSE87211, GSE190826, GSE119409 and WCHSC were normalized by using the rank-in algorithm (19).

### Identification of prognosis-related genes

Univariable Cox regression analyses were applied to identify the prognosis-related genes. Univariable Cox regression analysis was performed to explore the potential confounders. We use three hypothesis testing methods (Wald.test, Logrank.test and likelyscore.test). Gene expression levels with p values less than 0.05 in the three hypothesis testing methods were considered significant. Subsequently, we shall include prognostic variables that exhibit statistical significance with p<0.05 from the univariate analysis into the ensuing analysis.

### Construction and validation of the prognostic signature

After screening with univariable analyses, elastic net was adopted to ascertain the optimal panel of prognostic genes (20). Compared with the LASSO algorithm used in previous studies, the elastic net has two advantages. It can select more features than the number of samples in the dataset, which is problematic when dealing with high-dimensional data. Additionally, if data contain a group of features that are highly correlated, the LASSO penalty is going to randomly choose one feature from this group, whereas the elastic net penalized model would tend to select all. The elastic net penalty overcomes these problems by using a weighted combination of the l_1 and l_2 penalties as represented by the following mathematical formula in **Equation 1**:


(1) $$\hat{\text{β}}=\text{arɡmin}(\sum_{\text{i}=1}^{\text{n}}{\left({\text{y}}_{\text{i}}-\sum_{j}^{\text{p}}\beta_{\text{j}}{\text{x}}_{\text{ij}}\right)}^{2}+{\text{λ}}_{1}{\left|\left|\text{β}\right|\right|}_{1}+{\text{λ}}_{2}{\left|\left|\beta \right|\right|}^{2})$$

The IRGPM was established with random forest using the Python package scikit-survival 0.17.2 on the GSE87211 cohort (21, 22). The expression status of 20 selected prognostic-related genes was used as the model input. The goal is to predict DFS time. We employed grid search to optimize the model’s hyperparameters for best performance, adjusting the number of trees, maximum depth, and minimum number of branch nodes with specific step sizes. We evaluated the model’s general performance using a stratified 10-fold cross-validation. For each patient, the model assigns a prognostic risk score. Using the X-tile program, we divided the patients into low-risk and high-risk groups. We then compared the DFS of rectal cancer patients in these groups using the Kaplan−Meier method and log-rank test with the survival R package (23). Data from the GSE190826 cohort and WCHSC cohort, were used to validate the prognostic risk model. Due to the absence of DFS data, GSE119404 cohort is excluded from the prognostic risk model validation.

### Exploration of the molecular mechanisms underlying the prognostic signature

The limma R software package was used to analyze differentially expressed genes (DEGs) between the low- and high-risk groups (24). We used the cluster Profiler R package to carry out gene set enrichment analysis (GSEA) and to identify significantly enriched pathways between the low- and high-risk groups (25). A P value<0.05 and |log2Fold Change| > 0.58 were regarded as the cutoff values for statistical significance.

### Evaluation of the TIME

To determine the relationship between the IRGPM and the TIME, we explored the abundance of immune cells and stromal cells between different groups. The stromal score, immune score and ESTIMATE score of each patient were calculated using ESTIMATE method (26). Their differences were compared using the Wilcoxon signed-rank test, and p<0.05 was considered to indicate significance. Then, we calculated the infiltration values for rectal cancer dataset samples based on 28 immune gene sets. Using single-sample gene set enrichment analysis (ssGSEA), the proportions of immune cells were quantified (27). The scores of immune cells in different groups are shown on multi-boxplots. We also made comparisons of immune checkpoint activation between the low- and high-risk groups by the ggpubr R package.

### Statistical analysis

Most statistical analyses were performed using R software (Version 4.1.3; R Foundation for Statistical Computing, Vienna, Austria). The cutoff points for age ranges were determined using the X-tile program (http://www.tissuearray.org/rimmlab/). The survival rate was plotted using the Kaplan−Meier method and analyzed using the log-rank test. P<0.05 was considered statistically significant. Graphs related to R statistical analyses were drawn using the ggplot2 package in R.

## Results

### Patient information

**Figure 1:**
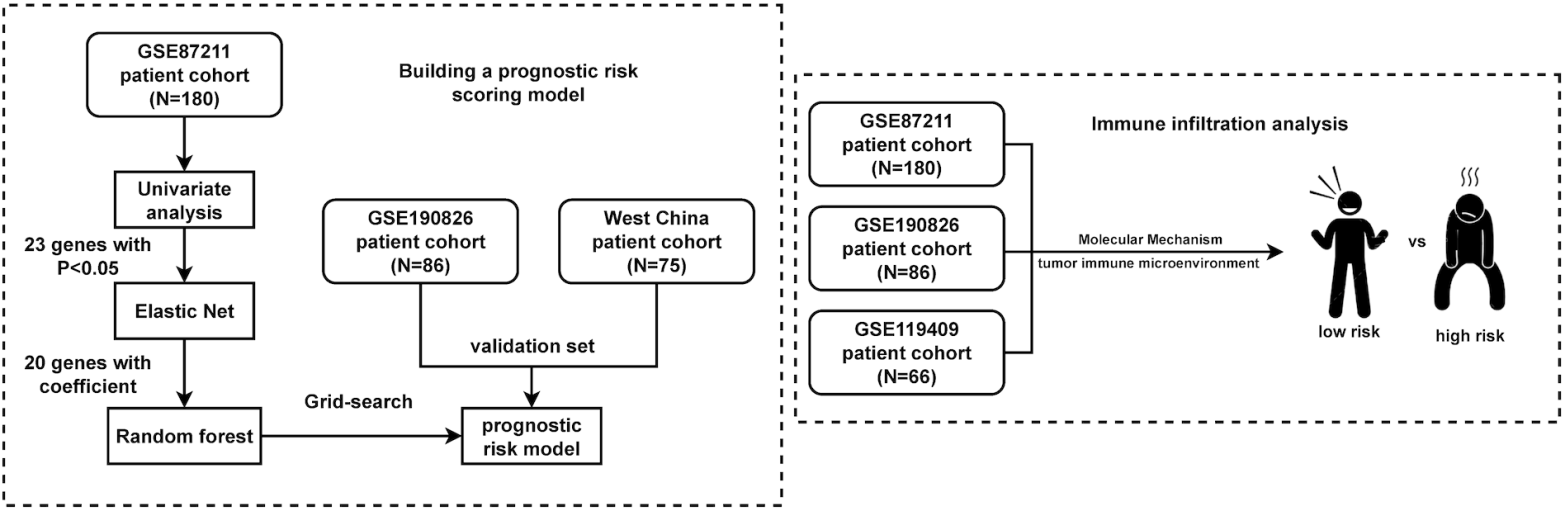
Flowchart of the study. A total of 407 LARC samples were used in this study. A training cohort (GSE87211) including 180 LARCs was used to identify a prognostic-risk class. Validation was then performed in 2 independent datasets. Immune analysis was performed in three cohorts of them.

The detailed flow diagram of our study is shown in **Figure 1**. A total of 407 LARC samples were used in this study. There were 180 LARC cases in the GSE87211 cohort that were used for prognostic risk model training. The GSE190826 dataset with 86 LARC cases and the WCHSC cohort with 75 LARC patients were used for model validation. For immune infiltration analysis, a total of 332 LARC samples in three GEO cohorts (GSE87211, GSE190826 and GSE119409) were included in the study. The detailed information (age, gender, tumor stage and treatment method) of patients from the GEO database and our hospital is shown in **Table 1**.

**Table 1:** Cohorts’ information in the study.

Dataset	GSE87211	GSE190826	GSE119409	SUWCH
Institution	National Cancer Institute, USA	Georg Speyer Haus, Germany	Peking University Cancer Hospital & Institute, China	Sichuan university, West China Hospital, China
No. of patients	180	86	66	75
Sex	Female:54 Male:126	Unknown	Unknown	Female:32(42.7%) Male:43(57.3%)
Age	≤65:73(40.5%) >65:107(59.5%)	Unknown	≤65:20(33.3%) >65:45(75%) Unknown:1(1.7%)	≤65:24(32%) >65:51(68%)
Clinical stage before neoadjuvant treatment*	II:59 III:117 IV:2 Unknown:2	II:6 III:57 Unknown:3	Unknown	II:9 III:66
Pathological stage after surgery*	pCR:68 I:17 II:47 III:45 IV:3	Unknown	Unknown	pCR:7 I:12 II:23 III:33
Treatment	nCRT	nCRT	nCRT	nCRT or SCRT

nCRT, neoadjuvant chemoradiotherapy; SCRT, neoadjuvant short course radiotherapy. *The staging is based on the 8th edition of the TNM staging system for colorectal cancer, as established by AJCC.

More detail are provided in **Supplementary Table S2**.

### Prognostic risk model construction

**Figure 2:**
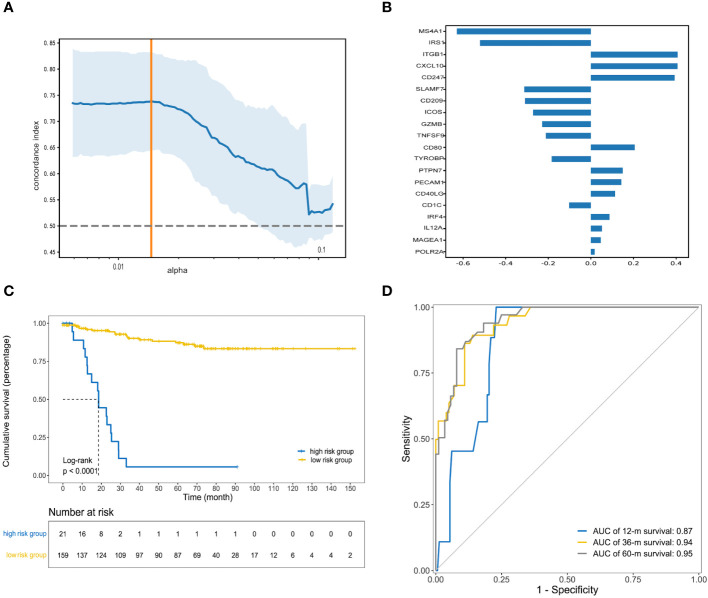
Construction of prognostic risk model. **(A)** The orange vertical line indicates the optimal value (c-index = 0.74), which was identified by tenfold cross-validation; **(B)** The random forest coefficient profiles of the 20 candidate genes; **(C)** Kaplan–Meier curve of DFS for patients with high and low prognostic risk scores in the training cohort; **(D)** Receiver operating characteristic (ROC) curve analysis for clinical benefit to preoperative chemoradiotherapy prediction. Area under the curve (AUC) estimation for the 20-gene signature in the training cohort.

To determine the independent prognostic genes, univariate Cox regression analyses for DFS were performed. As shown in **Supplementary Table S3**, 23 genes were identified and further included in the next analysis. To obtain the optimal panel, elastic net logistic regression was carried out on the candidate genes. We screened a 20-gene signature with the best concordance index (**Figure 2A**). Then, we constructed the optimized prognostic model by using the random forest method, which assigned each sample a prognostic risk score based on the identified coefficient of each gene (**Figure 2B**; details in **Supplementary Table S4**). To obtain the optimized cutoff value for the prognostic risk score, we applied the X-tile program to assess the statistical significance and avoid arbitrary cut point selection. The patients were then divided into high- and low-risk groups based on a cutoff value of 18.00. The Kaplan−Meier curves showed that the low-risk group was associated with a significantly longer median disease-free survival (mDFS) than the high-risk group (unreached vs. 20 months; p< 0.0001; hazard ratio (HR), 0.05; 95% confidence interval (95% CI), 0.03–0.11; **Figure 2C**). As shown in **Figure 2D**, receiver operating characteristic (ROC) analysis indicated that the areas under the curve (AUCs) of the IRGPM for the training cohort reached 0.87, 0.94, and 0. 95 at 1, 3, and 5 years, respectively.

### Validation of the prognostic risk model

**Figure 3:**
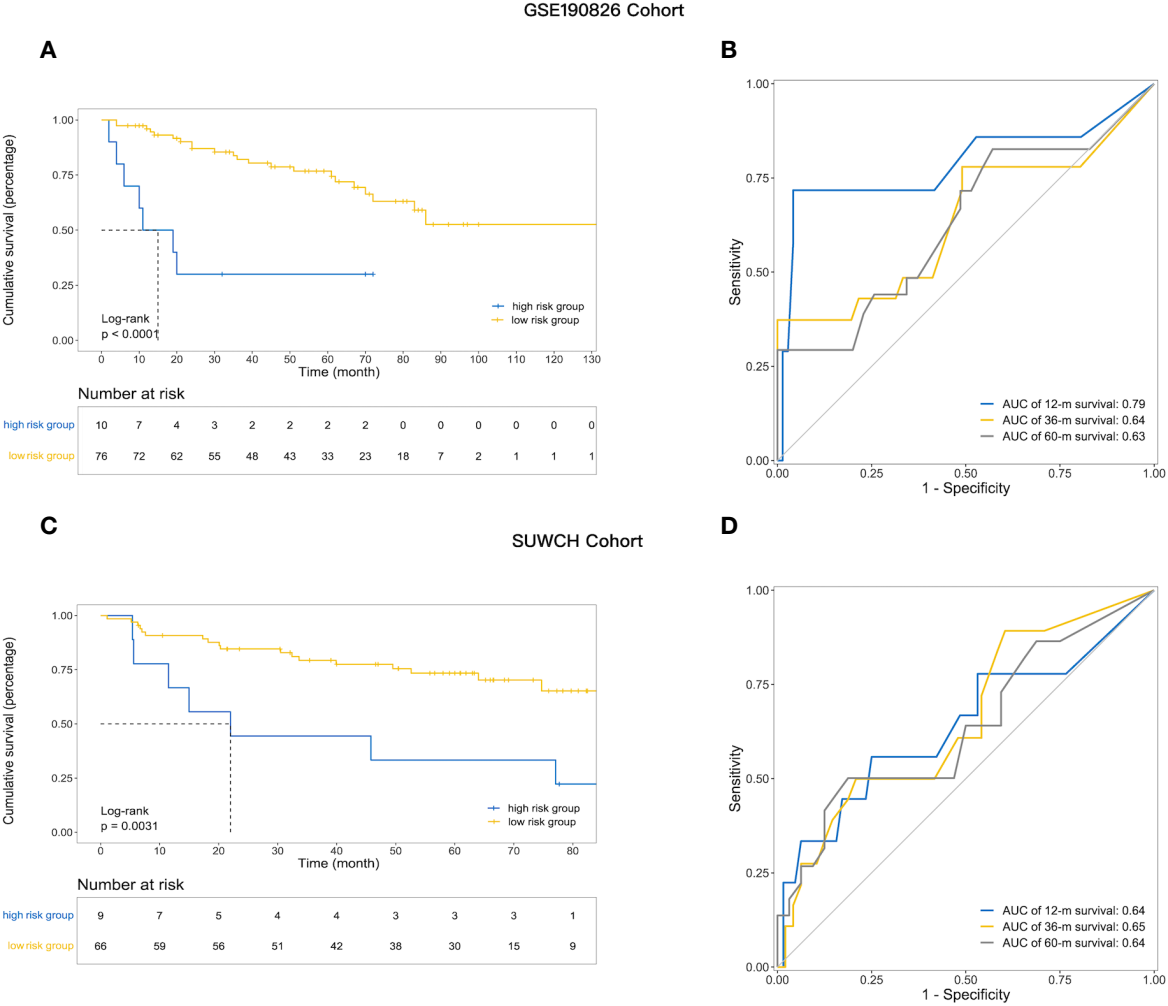
The utility of prognostic risk model in predicting the clinical outcomes of patients with LARC receiving neoadjuvant treatment. **(A)** Kaplan–Meier survival analysis of DFS among patients within each of the two indicated subgroups in the GSE190826; **(B) **The ROC curve analysis and AUC for the performance of prognostic model in the external validation set GSE190826; **(C)** Kaplan–Meier survival analysis of DFS in the SUWCH; **(D)** ROC curve and AUC in SUWCH cohorts.

The robustness of IRGPM was validated in two independent cohorts of LARC patients with adequate information on genomic expression profiles and survival. The patients in both cohorts were classified into high- and low-risk groups in the same way as the training cohort, with the optimal cut point determined in the training cohort (details in **Supplementary Table S4**). As shown in **Figure 3A**, for the GSE190826 cohort (n=86), low-risk patients (n = 76) had a better DFS than high-risk patients (n = 10) (mDFS, unreached vs. 13 months; p< 0.0001; HR, 0.52; 95% CI, 0.28–0.95). ROC analysis indicated that the AUCs of the IRGPM for the GSE190826 validation cohort were 0.79, 0.64, and 0.63 at 1, 3, and 5 years, respectively (**Figure 3B**). Consistently, in the SUWCH cohort (n=75), low-risk patients (n = 66) also had a better DFS than high-risk patients (n = 9) (mDFS, unreached vs. 22 months; p = 0.0031; HR, 0.29; 95% CI, 0.12–0.69; **Figure 3C**), and the AUCs for the prognostic risk model were 0.64, 0.66, and 0.64 at 1, 3, and 5 years, respectively (**Figure 3D**).

### Differential expression analysis

**Figure 4:**
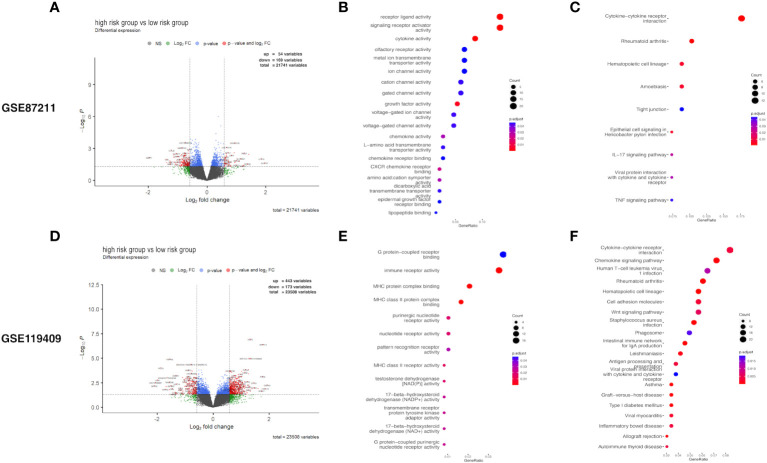
Differential expression analysis. Volcano plot of differentially expressed genes (DEGs) **(A)**, and the Gene Ontology (GO) enrichment **(B)** and KEGG enrichment **(C) **in GSE87211 cohort. DEGs identified **(D)**, and its GO enrichment **(E)** and KEGG enrichment **(F)** of identified DEGs in GSE119409 cohort.

The expression profiles of LARC patients with high prognostic risk scores were compared with those of patients with low risk scores to identify DEGs. The DEGs related to prognostic risk status for each cohort are listed in detail in **Supplementary Table S5** and visualized in volcano plots (**Figure 4**). In the GSE87211 cohort, a total of 54 upregulated genes and 169 downregulated genes were identified in the high-risk group compared with the low-risk group (**Figure 4A**). Functional enrichment analysis showed that the DEGs were significantly associated with 19 GO terms and 9 KEGG pathways (details in **Supplementary Table S6**). GO enrichment analysis of biological processes was conducted for the DEGs, which demonstrated that receptor ligand activity, signaling receptor activity, cytokine activity growth factor activity and CXCR chemokine receptor binding were the most frequently enriched biological process terms in the high-risk group (**Figure 4B**). The top enriched KEGG pathways by the DEGs in GSE87211 were also related to cytokine−cytokine receptor interactions (**Figure 4C**). In the GSE119409 cohort, 616 DEGs were identified, with 443 unregulated and 173 downregulated genes (**Figure 4D**). The functional annotation results showed that the DEGs were significantly associated with 13 GO terms and 30 KEGG pathways (**Supplementary Table S6**). The DEGs were mostly enriched in the GO terms MHC protein complex binding, immune receptor activity, MHC class II protein complex binding, and MHC class II receptor activity (**Figure 4E**). In addition, the most enriched immune-related KEGG pathways were cytokine−cytokine receptor interactions and chemokine signaling pathways (**Figure 4F**).

### GSEA of the DEGs

**Figure 5:**
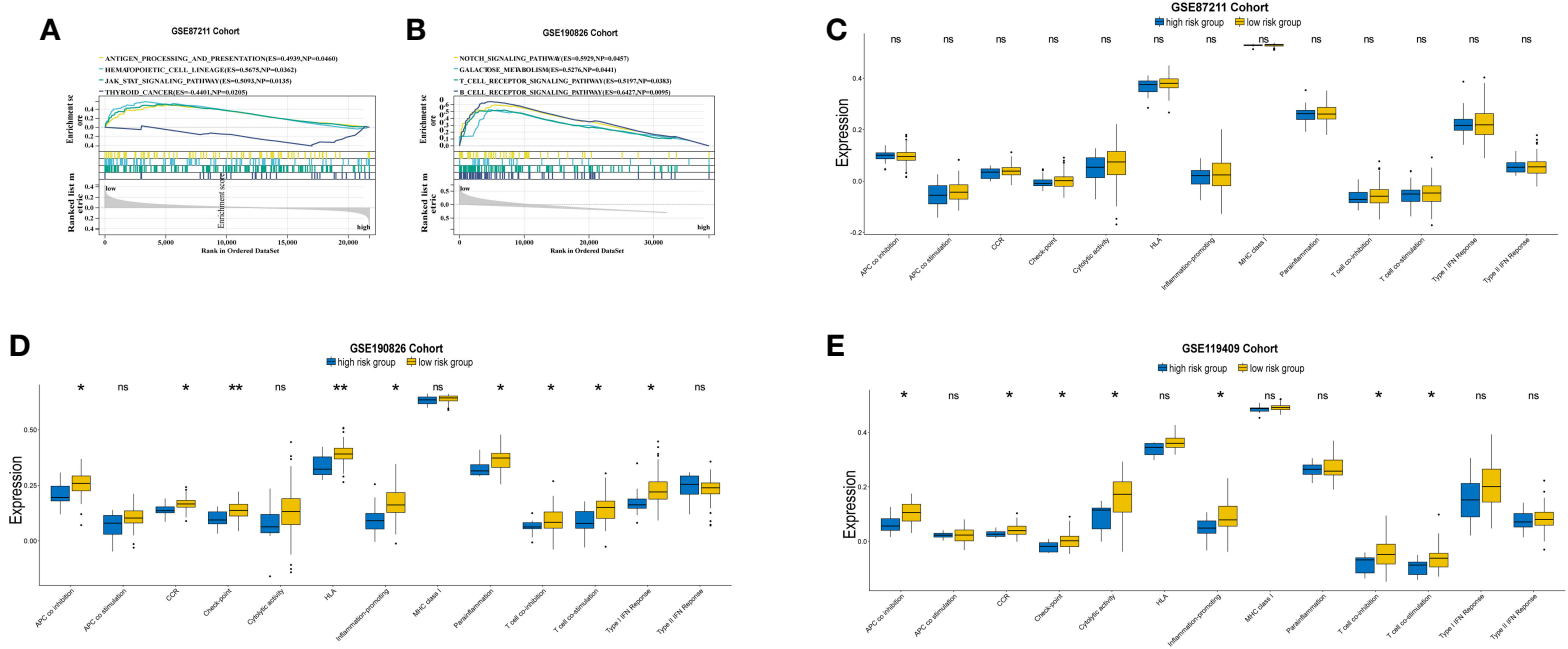
GSEA plot of immune-related and cancer-associated pathways in comparisons between the prognostic high and low risk groups in GSE87211 **(A)** and GSE190826 **(B)**.Comparations of 13 immune-related pathways expression between high and low risk groups in GSE87211 **(C)**, GSE190826 **(D)** and GSE119409 **(E)** cohorts. APC, Antigen-presenting cells; CCR, Chemokine receptors; IFN, interferon. *p<0.05; **p<0.01; ns, not significantly.

GSEA identified a number of KEGG pathways enriched in distinct prognostic risk subgroups (**Supplementary Table S7**). Typically, immune-associated pathways were highly active in the low-risk group, including antigen processing and presentation pathways, the hematopoietic cell lineage pathway, JAK–STAT signaling, T and B-cell receptor signaling, cytokine–cytokine receptor interactions, natural killer cell-mediated cytotoxicity and the intestinal immune network for IgA production (**Figures 5A, B**). In addition, we identified cancer-associated pathways that were hyperactivated in the low-risk group, including NOTCH signaling (**Figure 5B**). This suggests that the activities of these cancer-associated pathways are positively associated with LARC immunity. We also analyzed 13 immune-associated gene sets that represented diverse immune functions and pathways (**Supplementary Table S8**). The ssGSEA score was used to quantify the activity or enrichment levels of immune functions and pathways in the cancer samples. The gene sets of the low-risk samples were enriched in APC coinhibition, CCR, checkpoint, cytolytic activity, inflammation promotion, T-cell coinhibition, T-cell costimulation, type I IFN response and HLA (**Figures 5C–E**).

### Immune characteristics of different IRGPM subgroups

**Figure 6:**
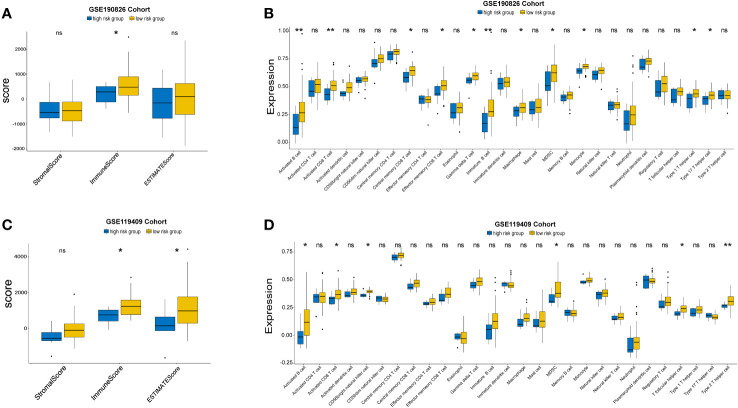
The Landscape of Immuno-cell Infiltration in the TME of LARC. The comparation of estimate, immune and stromal scores of high and low risk groups in GSE190826 **(A)** and GSE119409 cohort **(C)**. The expression of tumor-infiltrating immune cells in two subgroups in GSE190826 **(B)** and GSE119409 **(D)** cohort. The statistical difference of two groups was compared through the Kruskal-Wallis test and student-t test. *p< 0.05; **p< 0.01. ns, not significantly.

Given that the expression of immune genes is closely related to prognosis, it is necessary to investigate the role of the TIME in different groups. We compared the ESTIMATE scores, stromal scores, and immune scores between the high-risk group and the low-risk group. Although no significant differences were found in the GSE87211 cohort (**Supplementary Table S9**), low-risk patients had higher immune scores than high-risk patients in the GSE190826 cohort (**Figure 6A**), and the GSE119409 results showed that low-risk patients had higher ESTIMATE and immune scores than high-risk patients (**Figure 6C**). We then investigated 28 immune-associated gene sets that represented diverse immune cell types, and the ssGSEA score was used to quantify the activity or enrichment levels of immune cells. The low-risk group had higher levels of activated B cells, activated CD8 T cells, central memory CD8 T cells, effector memory CD8 T cells, gamma delta T cells, immature B cells, MDSCs, monocytes, type 1 helper cells and type 17 helper cells in GSE190826 (**Figure 6B**). Similarly, the low-risk group in GSE119409 had higher levels of activated B cells, activated CD8 T cells, CD56 bright natural killer cells, MDSCs, T follicular helper cells and type 2 helper cells (**Figure 6D**). The low-risk group had higher levels of activated CD4 T cells and type 2 helper cells in the GSE87211 cohort (**Supplementary Table S9**).

**Figure 7:**
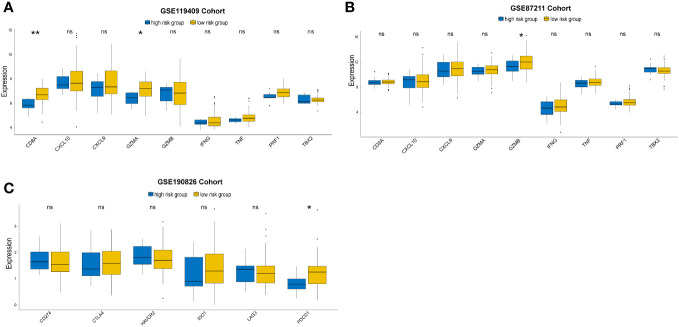
Immune-activation-relevant genes (CD8A, CXCL10, CXCL9, GZMA, GZMB, PRF1, IFNG, TBX2, and TNF) and immune-checkpoint-relevant genes (IDO1, CD274, HAVCR2, PDCD1, CTLA4, TIGIT and LAG3) expressed in high and low prognostic risk subgroups. The statistical difference of two groups was compared through the Kruskal-Wallis test. *p< 0.05; **p< 0.01. ns, not significantly.

We further investigated the expression of immune-activity-related genes, including CD8A, CXCL10, CXCL9, GZMA, GZMB, IFNG, PRF1, TBX2, and TNF. We found that the expression levels of CD8A,GZMA and GZMB were higher in the low-risk group than in the high-risk group (**Figures 7A, B**). In addition, we examined the expression of immune checkpoint genes related to the treatment response to immune checkpoint inhibitors in the two distinct subgroups. The expression status of seven genes previously identified as targets of immune checkpoint inhibitors were evaluated: CD274, CTLA4, LAG3, PDCD1, TIGIT, IDO1 and HAVCR2. The low-risk group had higher levels of PDCD1 than the high-risk group in GSE190826 (**Figure 7C**).

Based on the above analyses, we find that patients in low risk group had distinct TIME characterization compared with high risk group. Low risk group was characterized by adaptive immune cell infiltration and immune activation.

## Discussion

Due to the heterogeneity of tumors, even after standard treatment, including neoadjuvant chemoradiotherapy, over 30% of patients experience recurrence within 5 years (28). Consequently, it is an urgent task to develop reliable biomarkers that could identify patients with a high risk of recurrence after the standard treatment method. Then, intensive or different treatment schemes, as well as different monitoring schemes, can be appropriately selected for these patients. A previous study reported that immune-related genes can be used to predict the response to neoadjuvant therapy (15). However, the relationship between immune-related genes and the long-term prognosis (DFS) of LARC patients receiving neoadjuvant treatment followed by TME and adjuvant chemotherapy is currently unclear. We used a machine learning strategy to identify a 20-immune-gene signature that can predict the prognosis of LARC patients after nCRT. The prognostic model showed excellent discrimination for the individualized prediction of DFS and had an AUC of 0.95 for the prediction of DFS at 5 years.

The predictive model constructed in this study demonstrates robust predictive performance (AUCs of 0.94 in this study) (29–31). The good performance could be partly explained by the use of different machine learning classification algorithms in our study. Compared with the LASSO Cox regression analysis used in previous studies, the elastic net used in this study can select more features. Additionally, when data contain a group of features that are highly correlated, the elastic net penalized model tends to select all features, and the LASSO penalty randomly chooses one feature from this group. Therefore, the elastic net is more likely to choose the optimal panel for constructing a prognostic model. A previous study also showed that random forest and elastic net logistic regression were better than other studied classifiers (decision tree, neural network, and SVM) in treatment response prediction (18). In this study, with immune-related genes selected by elastic net, we applied random forest to construct the prognostic classifier of LARC. As demonstrated in the **Supplementary Table S11**, consistent with previous study outcomes, the model constructed via random forest exhibited superior performance.

To explore whether the immunological biomarkers at the RNA level could predict the prognosis of rectal cancer, an RNA immune-oncology panel was selected because it covered the signaling pathways of immunological system activation, tumor immune response, immune cell differentiation, immune regulation, tumor antigens, antigen presentation, and so on (32, 33). Previous studies have shown that it is possible to efficiently and accurately investigate, analyze and identify prognostic markers related to immune factors in the tumor microenvironment with an RNA immune-oncology panel (33, 34). Thus, an RNA immune-oncology panel was used to identify biomarkers in this study. In addition to the gene expression data from the database, we also detected the expression of 395 genes in the RNA immune-oncology panel at the RNA level from FFPE tumor specimens at WCHSC. The data originating from our institution were included in the validation cohort. Kaplan−Meier survival analysis showed poorer 5-year DFS in the high-risk group than in the low-risk group, and the ROC curve suggested good model prediction (AUC=0.64 at 5 years). This further proved that the developed prognostic risk model had good predictive power.

The immune gene signature in the prognostic model consisted of 20 genes, of which 11 genes were associated with T-cell immunity. TNFSF9, CD1C and CD209 play an important role in presenting antigens for T cells (35–37). ICOS, CD247, CD80, CD40LG and GZMB are associated with T-cell activation (38). ICOS, CD247 and CD80 belong to the CD28 family, which is critical for controlling the adaptive arm of the immune response (39). It is suggested that immunity has an effect on prognosis. CXCL10 is an interferon-stimulated chemokine that attracts T cells (15). IL12A and IRF4 are associated with T-cell differentiation (40). Five of 20 genes were shown to be predictive in rectal cancer in previous studies. CXCL10, IL12A, CD247 and ITGB1 were identified as predictive markers for the response to neoadjuvant treatment in rectal cancer in previous studies (15, 41–45). Tumor IRS1 expression was related to the survival of rectal cancer patients (46).

To further understand the immunologic properties of subgroups classified according to the prognostic model, we studied the characteristics of the TIME of the different subgroups. Through the ESTIMATE algorithm, we found that the immune score in the low-risk group was significantly higher than that in the high-risk group, suggesting that the infiltration of immune cells was significantly different between the two groups. Our data revealed a significant difference in the distribution of some immune cells between the high-risk group and the low-risk group in the GSE119409 and GSE190826 cohorts. In the low-risk group, activated B cells, activated CD8 T cells, central memory CD8 T cells, macrophages, T follicular helper cells, and type 2 helper cells were significantly increased compared with those in the high-risk group. This is consistent with the current understanding that the presence of abundant immune cells in TIME was associated with good prognosis (47). A previous study indicated that the presence of B cells was associated with successful tumor regression following nCRT in LARC (48). Shinji e et al. reported that a high density of CD8+ T cells in tumors in baseline biopsy samples was associated with a good response to treatment (7). It was revealed that macrophages, T follicular helper cells, and type 2 helper cells were related to the prognosis of rectal cancer patients (49). However, these results could not be confirmed in GSE87211. The inconsistencies might be due to tumor heterogeneity, different detection methods and the complexity of the TIME.

There were several limitations in our study that should be acknowledged. Our study was based on publicly available datasets, and it was not possible to obtain complete clinical information and demographic data for each patient. In addition, the potential mechanisms, molecular interactions and clinical applications of the prognostic genes of rectal cancer need to be further explored. The association between better TRG and favorable clinical outcomes continues to be a matter of debate (50). In this study, uncertainty remains regarding the relationship between the low-risk group characterized by a high immunoscore and TRG 0-1. Due to the absence of TRG staging data in the included database, we did not investigate the potential correlation between the low-risk group and favorable TRG regression in this study. Nevertheless, the exploration of predictive models for TRG remains critically important, and we plan to conduct further research in this domain in the future.

## Conclusions

In conclusion, this study developed a prognostic risk prediction model by analyzing immune-related genes and using the random forest and elastic net methods. The IRGPM has good prediction accuracy and provides a good stratification strategy for further trials. Different groups separated by IRGPM had distinct TIME characterization. These findings suggest that the immune response might play a vital role in the prognosis of chemoradiotherapy.

## Data availability statement

The original contributions presented in the study are included in the article/**Supplementary Material**, further inquiries can be directed to the corresponding author/s.

## Ethics statement

The studies involving humans were approved by Biomedical Ethics Review Committee of West China Hospital of Sichuan University. The studies were conducted in accordance with the local legislation and institutional requirements. The participants provided their written informed consent to participate in this study.

## Author contributions

PS: Data curation, Writing – original draft, Writing – review & editing, Conceptualization, Supervision. NL: Data curation, Writing – original draft, Writing – review & editing, Methodology, Validation. XL: Data curation, Methodology, Writing – original draft, Writing – review & editing, Visualization. YT: Conceptualization, Data curation, Writing – review & editing. ZC: Writing – review & editing. DL: Data curation, Writing – review & editing. DM: Methodology, Writing – review & editing. JD: Writing – review & editing. OY: Writing – review & editing. LS: Writing – review & editing. GO: Writing – review & editing. SW: Writing – review & editing. DJ: Writing – review & editing. XD: Writing – review & editing. ZW: Writing – review & editing. QL: Writing – review & editing. XW: Writing – original draft, Writing – review & editing.
